# Impacts of a Brazilian pharmaceutical program on the health of chronic patients

**DOI:** 10.11606/S1518-8787.2019053000733

**Published:** 2019-01-18

**Authors:** Aléssio Tony Cavalcanti de Almeida, Edvaldo Batista de Sá, Fabiola Sulpino Vieira, Rodrigo Pucci de Sá e Benevides

**Affiliations:** I Universidade Federal da Paraíba. Centro de Ciências Sociais Aplicadas. Departamento de Economia. João Pessoa, PB, Brasil; II Instituto de Pesquisa Econômica Aplicada. Diretoria de Estudos e Políticas Sociais. Brasília, DF, Brasil

**Keywords:** Chronic Disease, drug therapy, Pharmaceutical Services, Program Evaluation, National Policy of Pharmaceutical Assistance, Doença Crônica, tratamento farmacológico, Assistência Farmacêutica, Avaliação de Programas e Projetos de Saúde, Política Nacional de Assistência Farmacêutica

## Abstract

**OBJECTIVE:**

To evaluate the impact of the expansion of access to medicines by the *Programa Farmácia Popular do Brasil* (PFPB – Brazilian Popular Pharmacy Program) on the indicators of hospitalizations and deaths by hypertension and diabetes.

**METHODS:**

To estimate the impact of the Brazilian Popular Pharmacy Program, the statistical model of fixed-effect difference in differences was used, considering: the divisions *Rede Própria* (RP – Proprietary Network) and *Rede Conveniada* (RC – Partnership Network); the exposure time of the municipality to the program; intramunicipal density, measured by the number of accredited establishments; and the coverage spillover effect into patients from nonparticipating municipalities. Data from 5,566 municipalities were used, for the period from 2003 to 2016, including: (i) administrative records of the PFPB, *Sistema de Informações sobre Mortalidade* (SIM – Information System on Mortality), and *Sistema de Informações Hospitalares* (SIH – Hospital Information System); ii) other health data managed by the *Departamento de Informática do SUS* (DATASUS – Department of Informatics of SUS); iii) sociodemographic data produced by the Brazilian Institute of Geography and Statistics (IBGE); and iv) data from the *Relação Anual de Informações Sociais* (RAIS – Annual List of Social Information).

**RESULTS:**

The expansion of access to medicines for treatment of hypertension and diabetes resulted in a meaningful and statistically significant reduction (p < 0.05) of the number of hospitalizations and deaths by these diseases, in an average annual rate of 27.6% and 8.0%, respectively. The observed impacts were induced by the partnership network, highlighting the density of establishments per 100,000 inhabitants and, above all, the exposure time of the municipality to the program as relevant to the effect. Evidence of a spillover effect and of the maintenance of impacts on different age groups, especially older people, were also observed.

**CONCLUSIONS:**

The strategy to expand access to medicines through the PFPB was effective in reducing hospitalizations and deaths by hypertension and diabetes in Brazil during the investigated period. Better understanding the impacts of the program is important to improve the pharmaceutical care policy, to ensure access to cost-effective treatments.

## RESUMO

**OBJETIVO:**

Avaliar o impacto da ampliação do acesso a medicamentos pelo Programa Farmácia Popular do Brasil sobre indicadores de internações hospitalares e óbitos por hipertensão arterial e diabetes.

**MÉTODOS:**

Para estimar o impacto do Programa Farmácia Popular do Brasil, foi usado o modelo estatístico de diferenças em diferenças com efeito fixo, considerando: as vertentes rede própria e rede conveniada; o tempo de exposição do município ao programa; a densidade intramunicipal, medida pelo número de estabelecimentos credenciados; e o transbordamento espacial da cobertura em pacientes de municípios não participantes. Foram utilizados dados de 5.566 municípios, referentes ao período de 2003 a 2016, incluindo: i) registros administrativos do Programa Farmácia Popular do Brasil e do Sistema de Informações sobre Mortalidade e Sistema de Informações Hospitalares; ii) outros dados de saúde administrados pelo DATASUS; iii) dados sociodemográficos produzidos pelo IBGE; e iv) dados da Relação Anual de Informações Sociais.

**RESULTADOS:**

A ampliação do acesso aos medicamentos para tratamento da hipertensão e da diabetes resultou em redução expressiva e estatisticamente significativa (p < 0,05) do quantitativo de internações hospitalares e óbitos por essas doenças, em uma taxa média de 27,6% e 8,0% ao ano, respectivamente. Os impactos observados foram induzidos pela rede conveniada, sendo relevante para o efeito a densidade de estabelecimentos por 100 mil habitantes e, sobretudo, o tempo de exposição do município ao programa. Verificaram-se, ainda, evidências de transbordamento espacial e manutenção dos impactos sobre diferentes grupos etários, com destaque para os idosos.

**CONCLUSÕES:**

A estratégia de ampliação do acesso a medicamentos por meio do Programa Farmácia Popular do Brasil mostrou-se efetiva para a redução de internações e óbitos por hipertensão arterial e diabetes no Brasil no período investigado. Compreender melhor os impactos do programa é relevante para aperfeiçoar a política de assistência farmacêutica, de modo a assegurar o acesso a tratamentos com boa relação custo-efetividade.

## INTRODUCTION

In Brazil, chronic noncommunicable diseases (CNCDs) were responsible for 73% of deaths in 2016^1^. According to the Plan of Strategic Actions for Fighting CNCDs in Brazil from 2011 to 2022, hypertension and diabetes are the main causes of morbidity and mortality in the country, with high social and economic impacts^2^.

Pharmacological treatment integrated to primary health care is one of the most cost-effective ways to address cardiovascular diseases, including hypertension and diabetes. Currently, the Brazilian Unified Health System (SUS) ensures pharmacological treatment for these diseases in two ways: Basic Pharmacies, which are basic dispensing units of the Brazilian Unified Health System (SUS) in primary health care; and the PFPB.

The PFPB was thought of as a complement to the dispensation of medicines covered by Basic Pharmacies and operated in two divisions until 2017. The first was the Proprietary Network (RP), which came into operation in 2004 and was closed in 2017. It was managed by Fiocruz and included public pharmacies and drugstores installed by partnerships with municipal and state governments and nonprofit organizations to distribute medicines with copayment of a small portion or for free – which is the case of medicines for the treatment of diabetes, hypertension and asthma from 2011 on. The second is the Partnership Network (RC), which has been in operation since March 2006 and uses the capillarity of pharmacies from the private network to distribute medicines with copayment; since 2011, it also dispenses the medicines for hypertension, diabetes, and asthma for free.

Despite being implemented for over a decade, studies on PFPB are still scarce, and reviews about its impact on the health conditions of its beneficiaries are even more rare. Regarding impact evaluations, we highlight the study of Ferreira^3^, which uses municipal data from 2000 to 2012 to analyze the effects of distribution of medicines by the partnership network (PFPB-RC). The findings show the PFPB-RC reduces mortality rates by circulatory diseases and dyslipidemia, and that the addition of a partnership unit to every 100,000 inhabitants generates a reduction of 3.5 and 4.5 in the hospitalization rate for diabetes and hypertension per 100,000 inhabitants, respectively. The study also shows that the effects of the program increase with the age of the beneficiary, mainly from the age of 40.

Despite its importance, the analysis developed by Ferreira^3^ leaves open some relevant issues, such as the role of each part of the program and the heterogeneity of the effects over time, which can affect the conclusions on the PFPB effectiveness. Thus, this study aims to move forward on these issues, having as main objective to evaluate the role of expanding the supply of medicines by PFPB in its divisions RP and RC on indicators of hospitalizations and deaths by hypertension and diabetes. The specific analysis for these diseases is motivated by their importance among the major CNCDs, as well as by the fact that medicines for their treatment are included in the PFPB since its implementation in 2004, historically accounting for more than 70% of the spending with the program.

## METHODS

To estimate the PFPB impact, the statistical model of fixed-effect difference in differences was used, considering: the divisions of RP and RC; the exposure time of the municipality to the program; intramunicipal density, measured by the number of accredited establishments; and the coverage spillover effect.

Annual data from 5,566 Brazilian municipalities (99.9% of the municipalities in the country) were used. The initial research period is 2003, the year before the start of PFPB, and the final period was defined by data availability: 2016 for the analysis of the effects of the program on hospitalization rates and 2015 for mortality rates.

The outcome variables, rates of hospitalization and mortality by diabetes and hypertension per 100,000 inhabitants, were constructed from data of SIH and SIM, considering the codes of the International Classification of Diseases (ICD-10) related to the two diseases on the quantification of hospitalizations and deaths, in addition to the population estimates of IBGE. The explanatory variables of interest for this evaluation are related to the specificities of the program coverage. To better characterize this coverage, administrative data of the PFPB were used regarding participating establishments, such as location, date of accreditation, division (RP or RC), and monthly transfer flow of the program.

Thus, the impacts of the program were measured considering: 1) exposure time by type of division, defined by the amount of years in which the municipality had establishments of RC or RP with sales record of PFPB items; 2) coverage density, measured by the annual amount of establishments of the program per 100,000 inhabitants in each covered municipality; 3) the coverage spillover effect, measured by the number of municipalities, close to a noncovered municipality, that have establishments accredited to the program in a given period of time.

If, on the one hand, the exposure time captures the maturation of the PFPB impacts on the health status of the population, on the other, one must consider the density of coverage within the municipality. For example, there may be locations with identical times of exposure, but with different numbers of proprietary or partnership establishments per 100,000 inhabitants, and this difference affects the probability of access to medicine. Furthermore, considering that most of the Brazilian municipalities are small, the absence of PFPB in a given municipality does not necessarily implies that its residents cannot have access to medicines in neighboring municipalities covered by the program.

To better estimate the effects attributable to the PFPB, other explanatory variables were used, which can be responsible for the differences in results not directly related to the program and that can also affect the participation decision of establishments. These control variables increase the precision of the estimates of the program effects and also reduce possible selection biases^4^ ^,^ ^5^. Such biases are related to the nonmandatory character of the adherence to PFPB, so that locations with establishments of the program could present economic, social, demographic, and business characteristics different from those of the regions without accredited establishments. Thus, thinking about the eligibility criteria for adherence to the PFPB, variables that include population size (important for the RP, which was prioritized in places with greater population) and number of pharmacists (one of the criteria for accreditation) were used, while for the decision of accreditation of private establishments, commercial dimension proxies were also considered, such as size of the local market (given by the salaries of formal workers) and competition (total number of drugstores). Moreover, number of medical consultations in primary health care, number of hospital beds, number of higher education schools (a proxy for the education level of the municipality), and average income of formal workers were also included in the regression models, since they can affect the indicators of hospitalizations and deaths. All these variables are from DATASUS, RAIS, and IBGE.

In statistical terms, regression models with panel data were adjusted, using fixed effect and the difference in differences (DiD) estimator^6^ ^,^ ^7^. This approach allows one to control the non-observed time invariant heterogeneity, because municipalities covered by the program can be distinct from those not covered, and these differences can be correlated with the outcome indicators (hospitalizations and deaths).

Considering that individuals living in municipalities exposed longer to the PFPB, for having easier access, are more likely to adhere to the pharmacological treatment^7^, the basic specification of the model incorporates the scheduling of the coverage between the municipalities to measure effectiveness according to exposure time. It is also assumed that the municipal health indicators are mainly determined by demographic, social, and economic factors, as well as by health care, which includes pharmaceutical care^8^ ^,^ ^9^.

Equation 1 highlights the main model to evaluate the PFPB effects on population health indicators.

Equation 1 $$Y_{it,k}=\sum_{j=1}^{J}\beta_{j,k}RC_{j,it}+\sum_{g=1}^{G}\delta_{g,k}RP_{g,it}+\tau_{1,k}Estb_{it}+\tau_{2,k}NB_{it}+X_{it}^{'}\gamma_{k}+\phi_{i}+\mu_{st}+\epsilon_{it,k}$$

where *Y_it,k_* represents the indicator of result k for municipality *i* at time *t*, with k including the indicators of hospitalization and mortality; *RC_j,it_* is a binary variable that takes a value of one if municipality *i* in year *t* had any private establishment with sales record in the partnership network of the program for *j* years; *RP_g,it_* is a binary variable that takes a value of one if the municipality *i* in year *t* had some establishment of RP with sales record for *g* years; *Estb_it_* captures the number of establishments of the program per 100,000 inhabitants, given that the coverage density can vary over time; *NB_it_* measures its spillover effect by the number of neighbors of municipality *i*, in a radius of 50 km, covered by the PFPB at a time *t; X_it_* represents a vector of control variables of the municipalities; *ϕ_i_* is the fixed effect of the municipality, *μ_st_* represents temporal trends specific for the *s*-th federation unit of the municipality location; *ϵ_it,k_* is the random error term.

## RESULTS

Table 1 shows the estimated impacts of the PFPB on hospitalization and mortality by hypertension and diabetes. One can observe that the effects of the program on the two outcome variables are statistically significant (p < 0.05) only for RC. For example, in 10 years of exposure to this program division, the municipalities have reduced, on average, 100.3 hospitalizations per 100,000 inhabitants and 13.3 deaths per 100,000 inhabitants resulting from the two diseases.

**Table 1:** Estimated impact of the Brazilian Popular Pharmacy Program on the rates of hospitalization (2003–2016) and mortality (2003–2015) by diabetes and hypertension, according to the exposure time of the municipalities to the program and its divisions. Rates per 100,000 inhabitants.

Explanatory variables of interest		Dependent variables	
		Hospitalization	Mortality
Partnership network (RC)			
Exposure time			
	1^st^ year	-23.68* (273)	-2.89* (0.63)
	2^nd^ year	-29.87* (2.93)	-3.51* (0.68)
	3^rd^ year	-39.44* (3.20)	-3.30* (0.76)
	4^th^ year	-51.49* (3.48)	-3.61* (0.83)
	5^th^ year	-59.65* (3.80)	-6.50* (0.91)
	6^th^ year	-68.66* (4.16)	-7.25* (1.04)
	7^th^ year	-75.67* (4.66)	-8.81* (1.17)
	8^th^ year	-82.86* (5.23)	-9.58* (1.38)
	9^th^ year	-94.25* (6.13)	-10.33* (1.59)
	10^th^ year	-100.29* (7.05)	-13.31* (2.09)
	11^th^ year	-91.67* (9.25)	–
Proprietary network (RP)			
Exposure time			
	1^st^ year	17.33* (7.57)	1.15 (1.70)
	2^nd^ year	15.97* (7.61)	1.84 (1.72)
	3^rd^ year	8.03 (7.68)	1.68 (1.73)
	4^th^ year	5.30 (7.73)	1.70 (1.74)
	5^th^ year	4.89 (7.77)	3.17 (1.78)
	6^th^ year	3.90 (7.94)	2.97 (1.83)
	7^th^ year	4.12 (8.15)	2.92 (1.89)
	8^th^ year	3.90 (8.41)	3.46 (2.01)
	9^th^ year	3.50 (8.92)	4.44 (2.32)
	10^th^ year	1.19 (10.30)	5.53 (3.30)
	11^th^ year	12.47 (14.64)	9.99 (7.48)
	12^th^ year	28.42 (32.95)	12.07 (16.80)
	13^th^ year	3.45 (73.92)	
Coverage density (Estb)		-0.69* (0.06)	-0.01 (0.01)
Spillover effect (NB)		-7.91* (0.68)	-0.58* (0.16)
Tendency (*μ*)		X	X
Controls (*X*)		X	X
Fixed effect (*ϕ*)		X	X
Period (years)		14	13
Municipalities		5,566	5,566

* Value statistically different from zero (p < 0.05).

Note: Robust standard errors are clustered at the level of the municipality in parentheses. The tendency (*μ*) is represented by binary variables of years, specific to each Federative Unit. The controls (*X*) used were size of the population living in the municipality, number of pharmacists per 100,000 inhabitants, salaries of formal workers, number of pharmacies (total and per 100,000 inhabitants), medical consultations in primary health care per 100,000 inhabitants, number of hospital beds per 100,000 inhabitants, number of higher education facilities per 100,000 inhabitants, and average income of formal workers. The fixed effect (*ϕ*), one of the features of the panel data regression model, controls all unobservable factors invariant in time.

Coverage density is an important variable in the analysis. The increase of a partnership establishment by 100,000 inhabitants, by itself, is able to reduce on average 0.69 hospitalizations per 100,000 inhabitants, with an equivalent elasticity of −1.6%. That is, a magnification of 1% in the number of establishments in a municipality decreases, on average, the rate of hospitalization by hypertension and diabetes in 1.6%.

Additionally, the results show that municipalities without PFPB coverage that are neighbors of covered municipalities are also benefited, with an average reduction of 7.9 hospitalizations per 100,000 inhabitants and 0.6 deaths per 100,000 inhabitants, indicating spillover effect of the program coverage.

To detail the previous results, Tables 2 and 3 present the PFPB effects on hospitalization and mortality rates per 100,000 inhabitants, respectively, by age groups, diseases, and program divisions. Both tables do not show statistically significant effects (different from zero with a 5% significance level) of RP on the analyzed outcomes.

**Table 2:** Estimated impact of the Brazilian Popular Pharmacy Program on hospitalization rate, by age group, disease, and division of the program. Brazil, 2003–2016. Rates per 100,000 inhabitants.

Explanatory variables of interest		Age group (years)			Disease	
		26 to 39	40 to 59	60 or more	Diabetes	Hypertension
Partnership network (RC)						
Exposure time						
	1^st^ year	-2.13*	-8.20*	-12.20*	-3.34*	-20.33*
	2^nd^ year	-2.01*	-10.06*	-17.01*	-7.08*	-22.79*
	3^rd^ year	-2.78*	-13.53*	-22.11*	-10.72*	-28.72*
	4^th^ year	-3.53*	-18.59*	-27.65*	-16.92*	-34.57*
	5^th^ year	-3.72*	-20.97*	-32.79*	-21.67*	-37.98*
	6^th^ year	-4.65*	-24.33*	-37.62*	-26.30*	-42.36*
	7^th^ year	-4.94*	-27.85*	-40.91*	-30.15*	-45.52*
	8^th^ year	-5.02*	-29.95*	-46.06*	-36.91*	-45.94*
	9^th^ year	-4.07*	-34.56*	-54.22*	-42.83*	-51.41*
	10^th^ year	-5.27*	-36.80*	-56.85*	-46.37*	-53.92*
	11^th^ year	-3.63*	-36.12*	-50.67*	-42.04*	-49.63*
Proprietary network (RP)						
Exposure time						
	1^st^ year	2.21	5.69	6.99	1.64	15.68*
	2^nd^ year	1.76	5.22	7.41	2.07	13.90*
	3^rd^ year	0.44	1.97	5.01	1.93	6.1
	4^th^ year	-0.47	1.01	5.27	1.89	3.41
	5^th^ year	-0.7	1.28	5.39	1.89	2.99
	6^th^ year	-0.26	2.13	3.18	0.99	2.91
	7^th^ year	-0.22	1.58	4.08	1.35	2.77
	8^th^ year	-0.5	1.32	3.11	1.78	2.11
	9^th^ year	-0.44	1.06	3.93	1.59	1.91
	10^th^ year	-0.98	0.67	3.66	1.05	0.13
	11^th^ year	-0.69	6.88	8.78	1.48	10.99
	12^th^ year	-0.35	13.52	19.53	7.43	20.99
	13^th^ year	-3.41	-4.29	11.42	-0.73	4.18
Coverage density (Estb)		-0.03*	-0.26*	-0.43*	-0.37*	-0.32*
Spillover effect (NB)		-0.44*	-2.61*	-4.57*	-1.55*	-6.37*
Tendency (*μ*)		X	X	X	X	X
Controls (*X*)		X	X	X	X	X
Fixed effect (*ϕ*)		X	X	X	X	X
Period (years)		14	14	14	14	14
Municipalities		5,566	5,566	5,566	5,566	5,566

* Value statistically different from zero (p < 0.05).

Note: Robust standard errors are clustered at the level of the municipality in parentheses. The tendency (*μ*) is represented by binary variables of years, specific to each federation unit where the municipality is located. The controls (*X*) used were size of the population living in the municipality, number of pharmacists per 100,000 inhabitants, salaries of formal workers, number of pharmacies (total and per 100,000 inhabitants), medical consultations in primary health care per 100,000 inhabitants, number of hospital beds per 100,000 inhabitants, number of higher education facilities per 100,000 inhabitants, and average income of formal workers. The fixed effect (*ϕ*), one of the features of the panel data regression model, controls all unobservable factors invariant in time.

**Table 3:** Estimated impact of the Brazilian Popular Pharmacy Program on death rate, by age group, disease, and division of the program. Brazil, 2003–2016. Rates per 100,000 inhabitants.

Explanatory variables of interest		Age group (years)			Disease	
		26 to 39	40 to 59	60 or more	Diabetes	Hypertension
Partnership network (RC)						
Exposure time						
	1^st^ year	-0.10	-0.33	-2.28*	-1.18*	-1.71*
	2^nd^ year	-0.12	-0.40	-2.92*	-1,62*	-1.88*
	3^rd^ year	-0.23*	-0.20	-2.76*	-1.33*	-1.97*
	4^th^ year	-0.15	-0.71*	-2.49*	-1.50*	-2.11*
	5^th^ year	-0.23*	-0.88*	-5.28*	-3.50*	-3.01*
	6^th^ year	-0.31*	-1.01*	-5.89*	-3.95*	-3.29*
	7^th^ year	-0.31*	-1.20*	-7.26*	-4.90*	-3.90*
	8^th^ year	-0.39*	-1.42*	-7.50*	-6.14*	-3.44*
	9^th^ year	-0.34	-1.84*	-7.98*	-6.11*	-4.22*
	10^th^ year	-0.47	-2.18*	-9.93*	-8.44*	-4.87*
Proprietary network (RP)						
Exposure time						
	1^st^ year	0.10	0.09	0.33	1.00	0.15
	2^nd^ year	0.17	0.53	0.75	1.43	0.41
	3^rd^ year	0.02	0.19	0.89	1.17	0.51
	4^th^ year	-0.002	0.31	0.53	1.57	0.13
	5^th^ year	-0.01	0.95	2.19	2.05	1.11
	6^th^ year	0.07	0.61	1.15	1.94	1.03
	7^th^ year	0.07	0.69	1.76	2.10	0.82
	8^th^ year	0.05	0.63	2.66	2.38	1.09
	9^th^ year	0.09	0.83	2.95	3.14*	1.30
	10^th^ year	-0.004	1.33	4.15	2.97	2.56
	11^th^ year	0.22	0.82	6.56	5.85	4.13
	12^th^ year	0.20	3.68	14.02	6.66	5.41
Coverage density (Estb)		0.0002	0.001	-0.02	-0.02	0.002
Spillover effect (NB)		-0.03	-0.09	-0.46*	-0.06	-0.53*
Tendency (*μ*)		X	X	X	X	X
Controls (*X*)		X	X	X	X	X
Fixed effect (*ϕ*)		X	X	X	X	X
Period (years)		13	13	13	13	13
Municipalities		5,566	5,566	5,566	5,566	5,566

* Value statistically different from zero (p < 0.05).

Note: Robust standard errors are clustered at the level of the municipality in parentheses. The tendency (*μ*) is represented by binary variables of years, specific to each federation unit where the municipality is located. The controls (*X*) used were size of the population living in the municipality, number of pharmacists per 100,000 inhabitants, salaries of formal workers, number of pharmacies (total and per 100,000 inhabitants), medical consultations in primary health care per 100,000 inhabitants, number of hospital beds per 100,000 inhabitants, number of higher education facilities per 100,000 inhabitants, and average income of formal workers. The fixed effect (*ϕ*), one of the features of the panel data regression model, controls all unobservable factors invariant in time.

Considering the two diseases, the effectiveness of RC on the reduction of hospitalizations is higher for the older age groups; is more significant for hypertension than for diabetes; and grows according to the exposure time of the municipalities to the PFPB (Table 2). In 2016, for municipalities covered for 11 years, the program was able to reduce 50.7 hospitalizations per 100,000 inhabitants for individuals with 60 years or more and living in the covered municipalities (for the two diseases). In all age groups, the reduction was of 49.6 hospitalizations per 100,000 inhabitants for hypertension.

Regarding mortality, the effectiveness of the program is lower (Table 3). The reduction in the mortality rate among individuals with 60 years or more was of 9.9 deaths per 100,000 inhabitants in the municipalities covered for more time (tenth year, from 2006 to 2015) by RC. However, unlike what was observed for hospitalizations, considering all age groups, the reduction of the mortality rate was more significant for diabetes than for hypertension, with a decrease of 8.4 deaths against 4.9 deaths per 100,000 inhabitants in the same year.

The PFPB spillover effect is also statistically significant for the estimated mortality reduction coefficients: in the case of hypertension, for all age groups; in the case of diabetes, only for older people. Regarding hospitalization, municipalities without program coverage, but neighbors of covered municipalities, can reduce the hospitalization rate in all age groups and for both diseases.

The Figure summarizes the marginal effect of the PFPB over time, considering the estimates of Tables 1, 2, and 3 with 5% statistical significance level.

**Figure:**
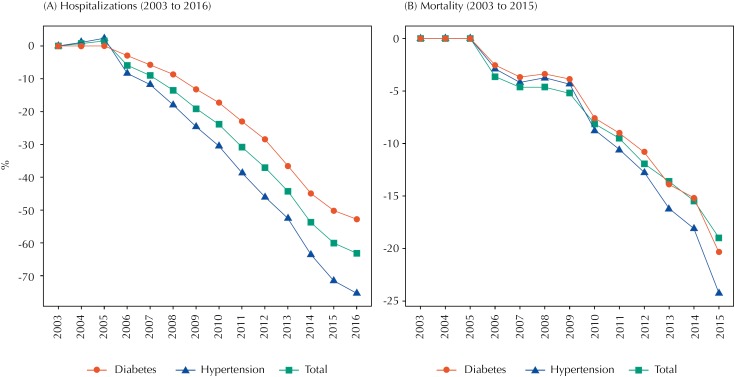
Rate of change of hospitalizations (a) and deaths (b) after implementation of the Brazilian Popular Pharmacy Program, total and by disease. Brazil, 2003–2016. Developed estimates based on coefficients of Tables 1, 2, and 3, considering a 5% statistical significance level and the overall effect of the program on the three aspects evaluated (exposure time, coverage density, and spillover effect).

The effectiveness indicator is the percentage reduction rate of hospitalizations and deaths by hypertension and diabetes. The PFPB was able to reduce the number of hospitalizations and deaths in SUS, respectively, at 27.6% and 8.0% per year, on average, for the period investigated. By the disease-specific estimates, we observed a similar behavior on the trajectory of the program effectiveness. For hypertension, the annual average reduction of hospitalizations and deaths was respectively of 33.7% and 8.8%, and for diabetes, 21.8% and 7.5%, during the period of analysis.

## DISCUSSION

Although initially designed as a complement to the dispensation of continuous-use medicines in pharmacies of SUS primary health care services, the demand for medicines and the expense with PFPB have quickly grown in recent years. In 2016, the expenses with PFPB were over 2.7 billion reals, which corresponds to more than double the amount spent by the Ministry of Health with medicines dispensed in primary health care^10^. The list of the program includes medicines for treatment of several CNCDs, but more than 71% of the resources were spent on hypertension and diabetes drugs that year.

Some studies have shown that the costs of the provision of medicines in SUS pharmacies may be lower than by the PFPB^11^ ^,^ ^12^. In this scenario, questions on the effectiveness of the program become relevant. As a rule, the evidence presented here suggest that improvement of access to the medicines covered by the program affected the adherence to the pharmacological treatment, regardless of the age group. This confirms the importance of access to medicines for obtaining better results in health, enabling greater population survival and lower costs for the health system^13^ ^,^ ^14^.

Given that there are problems of access to medicines by SUS patients, regardless of age or health group or evaluated health condition^15^, the results indicate that the expansion of availability has resulted in a significant reduction of hospitalizations and deaths – on average 27.6% and 8.0%, respectively, in the period of analysis. This finding shows the importance of ensuring access to pharmaceutical products by the public sector. This is particularly relevant in Brazil, considering that families with less income proportionally spend more on healthcare than those with higher income, and spending on medicines have a major stake in these expenses^16^ ^,^ ^17^.

From the PFPB divisions, RC was responsible for the impacts observed, being relevant to the effect the exposure time of the municipalities to the program and the density of establishments per 100,000 inhabitants. There is also evidence of program spillover effect, which means that even the population of municipalities that do not have accredited pharmaceutical establishments benefit from the program by having access to medicines in neighboring municipalities.

Concerning the non-identification of impacts in RP, it is important to note that this finding may be related to its low capillarity (reduced coverage and density). In 2016, only 7.3% of the municipalities had pharmacies linked to RP, with less than one establishment per 100,000 inhabitants. On the other hand, RC was present in most municipalities, with 82% coverage and average density of 17 establishments per 100,000 inhabitants.

Another issue is that RP may have a lower availability of medicines for hypertension and diabetes compared to RC^18^. In addition, considering its low capillarity, from the methodological point of view, the evaluation of the effectiveness of RP would be more appropriate if done with users rather than municipalities.

Regarding the expressive effects induced by RC on hospitalization and mortality indicators, one observes this division presents high coverage on national territory – with high intramunicipal density –, variety of brands, and availability of medicines for users^18^. Since an expressive part of the demand is formed by SUS patients^19^ (about 70% in 2014), the PFPB may be filling a gap in the public system itself, because of problems with the provision of medicines by municipal and state governments^13^ ^,^ ^19^ ^,^ ^20^.

Using data from the 2013 *Pesquisa Nacional de Saúde* (PNS – National Health Survey), Costa et al.^21^ have shown that 35.9% of hypertensive and 57.4% of diabetic individuals have obtained at least one medicine from the PFPB, with emphasis on the participation of socioeconomically disadvantaged segments. In this sense, as RC leverages the structure and distribution logistics network of drug stores and pharmacies in retail, it manages to provide access to medicines to a large group of individuals with difficulties to get them from SUS pharmacies and, thus, makes the continued treatment of the studied chronic diseases more effective.

The underestimation of the program effects in the absence of the variable that captures the overflow of its coverage may be related to the contamination of the group of non-covered municipalities, since the people living there can access the medicines in PFPB establishments from other municipalities. Considering that Brazil is formed by a large number of small municipalities (about 70% have less than 20000 inhabitants), and that there is a growing trend of commuting, it is likely that there is a potential demand for medicines and other items provided in neighboring municipalities. The inclusion of this variable in the model proved to be important.

The results of this study confirm the findings of Ferreira^3^, to the extent that the estimated coefficients in both studies have the same sign. The previous study identified as effect of the PFPB-RC the reduction of 3.5 and 4.5 hospitalizations per 100,000 inhabitants, respectively, for diabetes and hypertension. However, the coefficients shown in Table 2 of this article, as a whole, are much larger (in absolute terms) and more robust, especially for individuals aged 40 years or more, confirming the need to consider the time of exposure to continued treatment and to control the possible spillover effect of the program coverage. Comparing these findings, we concluded that the program was able to reduce, over time, more hospitalizations and deaths related to hypertension than diabetes.

In general, the findings show positive aspects of the program. However, even if its effectiveness is proven, the impacts of the replacement of pharmaceutical care for PFPB in primary health care must be investigated in further studies. Since most medicines of the program are on the *Relação Nacional de Medicamentos Essenciais* (RENAME – National List of Essential Medicines)^22^, it may encourage health secretaries not to buy them, especially at a time of economic recession and budget constraint, besides encouraging the suppliers not to take part in the bidding processes of prefectures, which have a higher risk of default, while the program involves a single payer^a^.

The limitations of this study include the fact that hospitalizations by hypertension and diabetes in the private health subsystem were not considered, which creates some uncertainty about the effectiveness of the program measured by the number of hospitalizations averted, unlike the effectiveness estimated as reduction of deaths, which is methodologically robust. Another limitation concerns the data used, which were not able to properly evaluate the effectiveness of RP, considering its low capillarity in the municipalities.

Finally, it should be noted that the analysis can be improved by future research. For instance, it would be possible to estimate disability-adjusted life years (DALYs), from data of individuals, and measure the effectiveness of the program by the number of averted DALYs. Based on this indicator and on information about the costs of the program, it would be possible to estimate its cost-effectiveness ratio and apply the parameter of the World Health Organization to evaluate the PFPB in this regard^23^ ^,^ ^24^.

## Footnotes

a Conselho Nacional de Secretários de Saúde. Apresentação das dificuldades enfrentadas pelas Secretarias Estaduais de Saúde na aquisição de medicamentos. Brasília (DF): CONASS; 2014 [cited 2018 Feb 1]. (Nota Técnica, 15). Available from: http://www.conass.org.br/biblioteca/wp-content/uploads/2014/01/NT-15-2014-atualizada-out.-DIFICULDADES-NA-AQUISIC%CC%A7A%CC%83O-DE-MEDICAMENTOS-NAS-SES.pdf
